# Proposed Cellular Function of the Human FAM111B Protein and Dysregulation in Fibrosis and Cancer

**DOI:** 10.3389/fonc.2022.932167

**Published:** 2022-07-04

**Authors:** Afolake Arowolo, Moses Malebana, Falone Sunda, Cenza Rhoda

**Affiliations:** ^1^Hair and Skin Research Laboratory, Division of Dermatology, Groote Schuur Hospital, Cape Town, South Africa; ^2^Department of Medicine, Faculty of Health Sciences, University of Cape Town, Cape Town, South Africa

**Keywords:** *FAM111B* gene, fibrosis, POIKTMP, cancers, protease

## Abstract

*FAM111B* gene mutations are associated with a hereditary fibrosing poikiloderma known to cause poikiloderma, tendon contracture, myopathy, and pulmonary fibrosis (POIKTMP). In addition, the overexpression of FAM111B has been associated with cancer progression and poor prognosis. This review inferred the molecular function of this gene’s protein product and mutational dysfunction in fibrosis and cancer based on recent findings from studies on this gene. In conclusion, FAM111B represents an uncharacterized protease involved in DNA repair, cell cycle regulation, and apoptosis. The dysregulation of this protein ultimately leads to fibrotic diseases like POIKTMP and cancers *via* the disruption of these cellular processes by the mutation of the *FAM111B* gene. Hence, it should be studied in the context of these diseases as a possible therapeutic target.

## Introduction

Mutations of the human *FAM111B* gene are also associated with a rare multisystemic fibrosing disease—poikiloderma, tendon contracture, myopathy, and pulmonary fibrosis (POIKTMP, which is the adopted terminology for this disease) (1–4). *FAM111B* gene mutations are also implicated in other clinical manifestations such as progressive osseous heteroplasia (POH) (5), autism spectrum disorders (6), modification of genes associated with cognitive development (7), nevus of Ota with choroidal melanoma (8), and mutations of unknown clinical significance/common genetic polymorphism (9). Furthermore, FAM*111B* gene mutations correlate positively with increased cancer predisposition (10–12). Moreover, the overexpression of this gene in cancer cells is associated with increased cancer progression and poor clinical outcomes (13–16). Although there are about 19 reported FAM111B mutations (**Supplementary Table 1**) to date (3–5, 10, 12, 17–24), the physiological function(s) of the FAM111B protein and its dysfunctional role in diseases like fibrosis and cancers are not well established.

This review highlighted the recent proposed cellular functions of the FAM111B protein to provide insights into the molecular basis of FAM111B protein dysregulation in POIKTMP and cancers.

## FAM111B as a Protease

Although the *FAM111B* gene products, specifically the protein, are not well characterized, various bioinformatics studies have predicted the presence of a putative trypsin/cysteine protease-like domain at the C-terminus of the FAM111B protein (2, 14, 25). The functional importance of this trypsin-like protease domain was demonstrated by Kawasaki et al. (14). A FAM111B protease domain-deletion construct failed to reduce cell proliferation in a *FAM111B* gene knockout cell line.

FAM111B is a paralog of FAM111A, the first member of this family of proteins, which also has this trypsin/cysteine-like protease domain (25, 26). FAM111A is a reported DNA-binding protein and plays an essential role in mitigating protein obstacles on replication forks by cleaving DNA-protein crosslinks (DPCs) (26, 27). The interaction of FAM111B with calpains (calcium-dependent cysteine proteases), specifically calpain small subunit 1 (CAPNS1) and calpain 1 (CAPN1), may also suggest the proteolytic function of FAM111B (12). FAM111B also shares about 45% sequence homology with FAM111A protein. However, both proteins’ three-dimensional (3D) structures are unavailable. Structural homology modeling using the *E. coli* DegS protease as a template has indicated shared structural homology most strikingly at the predicted catalytic domain (25), implying further the function of FAM111B as a protease.

## FAM111B in the Cell Cycle

**Figure 1:**
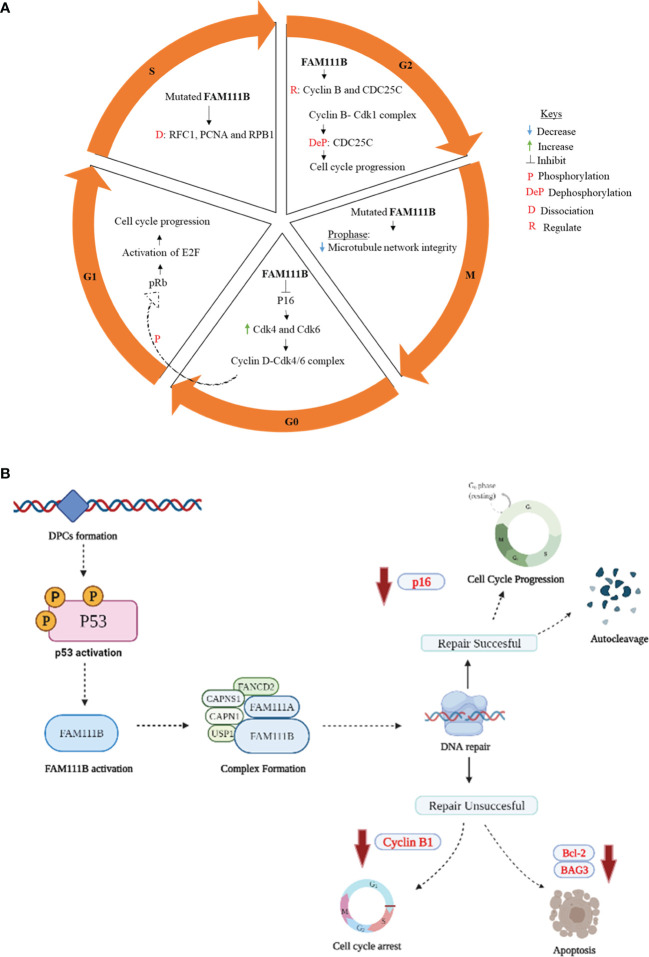
Proposed role of FAM111B in cell cycle regulation, DNA repair, and apoptosis. **(A)** G_0_/G1 phase: active p16 causes cell cycle arrest by inhibiting cyclin B1 and CDK. When FAM111B is activated, the protease degrades p16, allowing for cell cycle progression. S phase: mutations in FAM111B result in dissociation of RFC1, PCNA, and RPB1, which are involved in the synthesis of DNA. G2/M phase: FAM111B regulates cyclin B and CDC25C, allowing cell cycle progression to the M phase. M phase (prophase)- FAM111B mutations disrupt microtubule network integrity. **(B)** p53 is activated in response to DNA damage induced by DPCs formation. p53, in turn, directly or indirectly activates downstream signaling proteins, which eventually results in the activation of FAM111B. The active FAM111B, together with other DNA repair proteins such as FAM111A, responds by degrading the DPCs. FAM1111B will restart the cell cycle through p16 downregulation and autocleavage upon successful DNA repair. If the repair is not successful, FAM111B will induce apoptosis by downregulating the anti-apoptotic genes/proteins, e.g., BCL-2 and BAG3 (B created with Biorender.com).

One of the first published data supporting FAM111 B’s involvement in the cell cycle was from a multi-omics gene study, which showed the steady increase in FAM111B transcript levels during the G1 phase leading to detectable protein accumulation by the S phase (28). These results were further supported in a FAM111B-deleted lung adenocarcinoma (LUAD) cell line. A significant reduction occurred at the S and G2/M phases, with more cells remaining in the G_0_/G1 phases (14). This study suggested further that FAM111B degrades p16 (CDKN2A), a tumor-suppressor protein of the two classic cell cycle kinases: cyclin-dependent kinases 4 and 6 (CDK4 and CDK6) (29). These kinases form a cyclin D1-CDK4/6 complex which phosphorylates the retinoblastoma protein (pRb1) (14, 30). The phosphorylated pRb1 dissociates from E2F, enabling cell cycle progression from the G1 to S phase (31). Furthermore, a marked increase of p16 and phosphorylated pRb1 and E2F expression levels was reported in a FAM111B knockout cell line, leading to these cells’ lack of progression to S and G2/M and cell cycle arrest at the G_0_/G1 phase (14). Insights into the role of FAM111B protein during the S phase of the cell cycle were also provided by discovering the interaction between FAM111B and DNA-binding proteins like RFC1 (replication factor C subunit 1) and PCNA (proliferating cell nuclear antigen) (25). The RFC is a five-subunit protein that assists with loading PCNA into DNA, a crucial step in DNA replication (32). The disruption of these replication forks complex proteins: PCDNA and RFC were reported in FAM111B-dysregulated (overexpressed) and patient-derived mutant-expressing cells (25). Another study reported a strong association of FAM111B with G2/M phase proteins, precisely cyclin B1 (CCNB1), and the dual-specificity protein phosphatase CDC25C (13). For a successful G2/M transition, cyclin B must form a complex with CDK1 (a.k.a. CDC2 or p34). This complex is kept inactive by phosphorylation of CDK1 and activated by dephosphorylation CDC25C (33, 34). CDC25C also plays a crucial role in the cell cycle as a checkpoint protein, especially in DNA damage, thus ensuring accurate genomic information to daughter cells (35). Hence, a positive correlation of FAM111B with CCNB1 and CDC25C suggests that FAM111B promotes cell cycle progression into the mitotic phase. Moreover, mutations in the FAM111B gene were also shown to cause replication and transcriptional shutdown, disruption of microtubule network integrity at the M phase, and increased apoptosis (25). Hence, the proposed functions of FAM111B in cell cycle and division are hereby summarized in **Figure 1A**.

## FAM111B in DNA Repair and Apoptosis

Mutations in the *FAM111B* gene are associated with the loss of genome stability and integrity, suggesting its role in DNA repair and genome integrity (12, 36). The exact details of how FAM111B corrects the DNA are yet to be determined. However, given the possible functional overlap between FAM111A and FAM111B, we can hypothesize the possible mechanism of repair based on the known functions of FAM111A. The FAM111A, as a DNA-protein crosslink (DPC) protease, plays a role in the removal of DPCs that stall DNA replication (26, 36). DPCs occur when proteins within the cell become covalently trapped on a DNA strand due to exposure to endogenous/exogenous (e.g., formaldehyde) and chemotherapeutic agents. These DPCs interfere with DNA replication, repair, and transcription, eventually leading to genomic instability in the form of DNA double-strand breaks (36). FAM111A, among other DPCs, proteases, and possibly FAM111B are involved in the proteolytic cleavage of DPCs, hence forestalling DNA damage, genome instability, and DNA-damage-mediated apoptosis (25, 26, 36). Following the repair of DPCs, FAM111B and FAM111A should possibly undergo autocleavage like the essential mammalian DPC protease, SprT-like N-terminal domain (SPRTN) (37, 38). Although not well characterized, this regulatory process is thought to ensure the non-specific degradation or disruption of protein complexes crucial for maintaining genome integrity, such as the chromatin-associated RNA polymerase II (RPB1), PCDNA, or other histone proteins (25, 36, 39). Its interaction with CAPNS1 further suggests the role of FAM111B in DNA repair-CAPN1 [calpain small subunit 1 (Calpain 1)], USP1 (ubiquitin-specific protease 1), and FANCD2 (Fanconi anemia group D2 protein) (12). FAM111B interacts with CAPNS1, which forms a complex with CAPN1 (i.e., CAPNS1-CAP1) that stabilizes USP1 deubiquitinase, which, in turn, modifies FANCD2, an essential protein involved in various DNA damage response pathways (12, 40). Furthermore, the downregulation of antiapoptotic genes such as BCL-2 (B-cell lymphoma 2) and BAG3 (Bcl-2-associated athanogene 3) has been reported in FAM111B knockout cells (13, 25). Since there is evidence suggesting that FAM111B is involved in the DNA repair pathway and apoptosis (13, 25, 36), this finding begs the possible involvement of other DNA-damage-induced stress response pathways associated with FAM111B. The answer to the question was hinted at by Sun et al. (2020) by linking FAM111B to the p53 signaling pathway using KEGG enrichment analysis (13). The phosphorylation and activation of the p53 pathway are known to cause cell cycle arrest, cell senescence, or apoptosis in response to intracellular stresses such as DNA damage (41, 42). The *FAM111B* gene is reportedly enriched in the p53 signaling pathway, and the knockout of FAM111B reportedly affected the expression of *p53*-related genes such as *BAG3*, *BCL-2*, and *CCNB1 (* 13). Thus, it is firmly possible that FAM111B is part of the p53 signaling pathway. Interestingly, previous studies have indicated that the knockout of FAM111B does not affect the *p53* gene or protein expression (13, 14), suggesting that FAM111B operates downstream of p53. Hence, we propose that FAM111 B’s function in DNA repair and apoptosis is mediated by the direct or indirect downstream stream activation of FAM111B by the p53 (**Figure 1B**).

## FAM111B in Fibrosis: Hereditary Fibrosing Poikiloderma (POIKTMP)

*FAM111B* gene mutations are associated with POIKTMP, a hereditary multisystemic fibrosis disorder (2). This syndrome is characterized by fibrosis in multiple organs such as the skin and lungs (1, 2); however, the molecular basis of the FAM111B mutations in this disease remains unclear. Fibrosis results from excessive extracellular matrix component (ECM) deposition by activated fibroblasts when trigged by multiple stimuli (43). One such trigger is chronic from a sustained release of pro-inflammatory cytokines such as TGFβ and IL-13 (44). FAM111B and FAM111A gene mutations are suggested to result in the rapid cleavage of both proteins, possibly due to mutational gain of function of their protease activity (10). This rapid self-cleaving activity of these proteins, specifically FAM111B in POIKTMP, could result in the ineffective removal of DPCs or the disruption/degradation of PCNA-associated proteins like RFC1 and chromatin-bound RPB1 (36, 39), thus leading to failure in DNA repair and, ultimately, genome instability.

Furthermore, incomplete DNA repair and genome instability could result in excessive cell cycle arrest (given the regulatory role of FAM111B on *p16*) and ultimately apoptosis which may, in turn, trigger chronic inflammation due to the incomplete clearance of apoptotic cells (efferocytosis) by granulocytes (45), hence the chronic inflammation seen in the various tissues affected by POIKTMP. Furthermore, chronic inflammation is known to cause several types of alopecia, such as primary scarring alopecia (PSA) (12, 46), and may also explain the alopecia seen in patients with POIKTMP. Interestingly, mutations in DNA repair genes, genomic instability, and chronic inflammation play a significant role in other hereditary disorders like Hutchinson–Gilford Progeria Syndrome (36, 46, 47); Progeria causes clinical manifestations like POIKTMP as alopecia and joint contractures (46). This information further supports the view that genomic instability from *FAM111B* gene mutations causes POIKTMP (15, 36). However, the molecular mechanisms behind how *FAM111B* gene mutations lead to these diseases remain elusive.

Alternatively, fibrosis, a key clinical feature of POIKTMP, may also result from the failure of ECM-producing fibroblasts to undergo apoptosis after tissue remodeling. Resistance to apoptosis in idiopathic pulmonary fibroblast, alveolar macrophages, and impaired re-epithelization due to increased apoptosis of epithelial cells have been documented as the leading cause of collagen over deposition, further supporting this hypothesis (47–50). Moreover, bioinformatics and experimental studies imply high FAM111B mRNA and protein expressions in epithelial cells/tissues of cancer and non-cancerous tissues (13, 14, 51). Thus, as described previously, it is conceivable that the low expression or rapid degradation of FAM111B caused by the FAM111B mutations could lead to increased epithelial apoptosis and, consequently, the resistance to apoptosis of adjoining fibroblasts of the tissues affected by POIKTMP.

**Figure 2:**
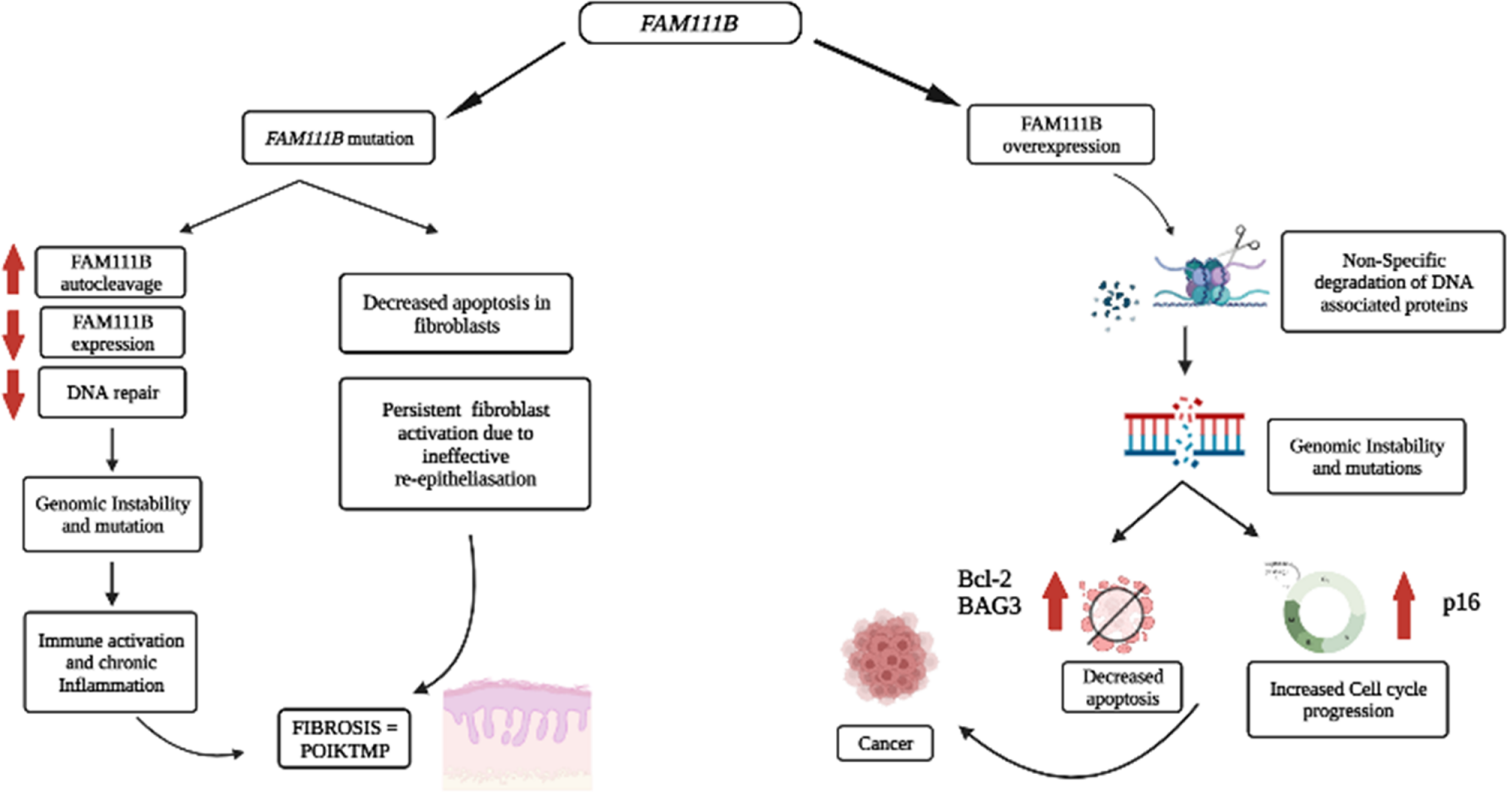
Proposed pathological processes involving FAM111B in cancer and POIKTMP. An increase in FAM111B expression possibly leads to an increase in proteolysis of DPCs and non-specific degradation of chromatin-associated proteins, which leads to genomic instability. Genomic instability is a significant characteristic of cancer. A decrease in FAM111B possibly leads to the reduction in apoptosis of fibroblasts, which causes an increase in fibrosis (Created with Biorender.com).

Therefore, we proposed that the mutational dysfunction of FAM111B protein causes POIKTMP by downregulating FAM111B gene and protein expression that are resulting in inadequate DNA repair, genome instability, chronic inflammation, and aberrant apoptosis of the epithelial cells and fibroblasts of the tissues/organs affected by this disease (**Figure 2**).

## Role of FAM111B in Cancers

The overexpression and mutations of the *FAM111B* gene are associated with several types of cancers, including pancreatic cancer, which happens to be one of the most life-threatening types of cancer (11, 12, 15). In addition, the overexpression of FAM111B in lung adenocarcinoma (LUAD) patients correlated strongly with increased tumor progression and poor survival rate (13, 14). There is, however, no evidence to support the degradation or clearance of endogenous FAM111B in cancers. Furthermore, multiple single-nucleotide polymorphisms (SNPs) associated with prostate cancer localize on chromosome 11q12, which houses the *FAM111B* and *FAM111A* genes (52). Given the possible involvement of FAM111B in DNA repair (5, 10, 29), the overexpression of the *FAM111B* gene can support cancer progression. One possible explanation could be that the overexpression of FAM111B results in the non-specific proteolytic degradation of other DNA-associated proteins such as histones and replication or transcription factors (e.g., RFC1 and RPB1) and cell-cycle-dependent proteins (e.g., p16). FAM111 B’s activity switches on in normal states upon detecting DPCs, perhaps through p53 signaling. The activation of FAM111B will then proteolytically cleave the DPCs to repair the DNA. However, once the repair is complete FAM111B may undergo autocleavage to prevent itself from cleaving other proteins involved in DNA metabolism and the cell cycle (29). Therefore, the overexpression of FAM111B and the non-specific degradation of DNA-associated proteins may cause genomic instability and dysregulation of the cell cycle, eventually contributing to cancer predisposition, development, and progression (**Figure 2**).

Furthermore, to salvage genome stability and the apoptotic fate of cells, the error-prone translesion (TLS) DNA polymerases are mobilized to replace conventional high-fidelity DNA polymerases, which can overcome the DPC barrier to DNA replication (36, 53). This DNA damage tolerance mechanism and the uncontrolled degradation of tumor-suppressor proteins, p16, could explain why cancer cells can evade apoptosis. Another possible explanation is that FAM111B/FAM111A-repair machinery confers some advantage to cancer cells at later stages of cancer, thus supporting cancer progression. For instance, in cervical cancer patients, FAM111B, among other genes, is only overexpressed in later stages of cancer, and it is associated with distal cancer progression and metastasis (26, 27, 36). Therefore, it is plausible that FAM1111 B’s upregulation is an adaptation strategy by cancer cells as most cancer drugs induce DNA damage by forming DPCs. In other words, FAM111B overexpression is an attempt to minimize genomic instability brought about by the formation of DPCs and conferring resistance to therapy.

## Discussions and Conclusions

Based on the published literature, one can conclude that the molecular functions of FAM111B include the response to DNA damage resulting from DPCs, which can stall DNA replication and cause breaks in the DNA strands and genome instability. The DPCs are possible triggers for p53 activation, activating the downstream signaling proteins, eventually starting FAM111B and FAM111A. These two proteins may function individually or by forming a complex with other DNA repair proteins (CAPNS1-CAPN1, USP1, and FANCD2) to degrade DPCs to facilitate DNA repair and resumption of DNA replication (**Figure 1**) (25). Furthermore, FAM111B may function with other DPC proteases, DNA repair, replication, and transcription proteins to restore genome stability, cell cycling, and turnover (25, 26, 36). If the repair is successful, FAM111B will relieve the cell cycle arrest by degrading p16 so that cell division can resume as normal. Also, FAM111B, as a possible regulated protease, should undergo autocleavage to prevent non-specific cleavage of chromatin-associated proteins such as histones and transcription factors (37). If the repair is not successful, FAM111B, among other proteins, triggers the expression of *BAG3* and *BCL-2* genes to induce apoptosis of the cell (**Figure 2**) (25).

In the case of the mutated form of this protein, which is commonly a gain of function, the proteolytic activity of FAM111B may be amplified, resulting in the non-specific cleavage chromatin-associated proteins, among others crucial. Additionally, other essential functions of FAM111B, such as activating apoptosis, may be lost. Finally, since FAM111B is critical for degrading p16 for cell cycle progression, the mutations may result in abnormal degradation of p16 and thus cause abnormal cell division (**Figure 1B**). As a result, FAM111B mutations tend to cause diseases such as POIKTMP and cancer through these mechanisms (**Figure 2**). More importantly, FAM111B seems more critical in some cell types than others. For example, the knockdown of FAM111B in Hela cells did not affect cell proliferation but did in lung adenocarcinoma cell lines. Thus, *FAM111B* mutations can affect certain cell types but spare the others (13, 14).

Although the pathological knowledge effects of the mutant forms FAM111B are emerging, only a handful of studies have investigated the physiological function of FAM111B (which remains unclear) in healthy cells/tissues. Therefore, the proposed functions may lack accuracy since most reported studies were conducted using cancer cells. Therefore, future studies to establish the molecular function of this protein should be conducted in both healthy and disease-relevant models.

In conclusion, the human FAM111B protein is crucial to specific cellular processes essential for cell viability or fitness. Therefore, it is imperative to study the dysregulation of this protein in the context of fibrosing diseases like POIKTMP and cancers. Moreover, FAM111B represents a promising therapeutic target for both disorders.

## Author Contributions

MM: writing and visualization; FS: writing and visualization; CR: writing; AA: conceptualization, writing, editing, and supervision. All authors contributed to the article and approved the submitted version.

## Funding

This work was supported by the South African National Research Foundation (NRF) Thuthuka Funding Instrument (NRF Rating Track) [grant number: TTK170413227114] and the South African Medical Research Council (SAMRC) [grant ref. number: HDID8682/MB2022], through its Division of Research Capacity Development under the Early Investigators Programme, from funding received from the South African National Treasury. The content hereof is the sole responsibility of the authors and does not necessarily represent the official views of the SAMRC or the funders.

## Conflict of Interest

The authors declare that the research was conducted in the absence of any commercial or financial relationships that could be construed as a potential conflict of interest.

## Publisher’s Note

All claims expressed in this article are solely those of the authors and do not necessarily represent those of their affiliated organizations, or those of the publisher, the editors and the reviewers. Any product that may be evaluated in this article, or claim that may be made by its manufacturer, is not guaranteed or endorsed by the publisher.

## Supplementary Material

Supplementary Table 1 List of reported FAM*111B* gene mutation and associated clinical phenotypes.
