# Supplementary material for: Proposed Cellular Function of the Human FAM111B Protein and Dysregulation in Fibrosis and Cancer
Source: Front Oncol. 2022 Jul 4;12:932167. doi: 10.3389/fonc.2022.932167 (PMC9293052; doi:10.3389/fonc.2022.932167)
Supplement: Supplementary Table 1 — List of reported FAM111B gene mutation and associated clinical phenotypes. [file Table_1.doc]

| **No.** | **FAM111B mutation** | **Mutation type/ Class** | **Clinical Phenotype** | **Reference** |
| --- | --- | --- | --- | --- |
| 1 | c.368_369insCT (p. ln124Phefs*15) | Non-synonymous insertion frameshift  Pathogenic? | Cognitive and developmental disorders | (7) |
| 2 | c.394T>C (p. Tyr132His) | Non-synonymous  Pathogenic? | Nevus of Ota choroidal melanoma | (8) |
| 3 | c.816dup (p. Ala273Serfs*9) | Frameshift duplication  Pathogenic? | Autism spectrum disorder | (6, 10) |
| 4 | c.917A>G (p. His306Arg) | Non-synonymous  Pathogenic-associated polymorphism | Systemic sclerosis-related | (9) |
| 5 | c.952G>T (p. Glu318Term) | Non-synonymous/truncation  Pathogenic | Colorectal cancer | (10) |
| 6 | c.988C>T (p. Leu330Leu) | Synonymous  polymorphism | Systemic sclerosis-related | (9) |
| 7 | c.1247T>C (p. Phe416Ser) | Non-synonymous  Pathogenic | POIKTMP | (46) |
| 8 | c.1261_1263delAAG (p. Lys421del) | In-frame deletion  Pathogenic | POIKTMP with Exocrine pancreatic dysfunction | (47) |
| 9 | c.1289A>C (p. ln430Pro) | Non-synonymous  Pathogenic | POIKTMP | (1, 48, 49) |
| 10 | c.1462delT (p. Cys488Valfs*21) | Frameshift deletion  Pathogenic | POH | (5) |
| 11 | c.1860T>G (p. Tyr621Asp) | Non-synonymous  Pathogenic | POIKTMP | (2) |
| 12 | c.1860T>G (p. Tyr621Asp) | Non-synonymous  Pathogenic | POIKTMP | (2) |
| 13 | c.1873A>C (p. Thr625Pro) | Non-synonymous  Pathogenic | POIKTMP | (50) |
| 14 | c.1874C>A (p. Thr625Asn) | Non-synonymous  Pathogenic | POIKTMP | (1) |
| 15 | c.1879A>G (p. Arg627Gly) | Non-synonymous  Pathogenic | POIKTMP | (2, 12, 51) |
| 16 | c.1881 A>T (p. Arg627Ser) | Missense | POIKTMP | (52) |
| 17 | c.1883G>A (p. Ser628Asn) | Non-synonymous  Pathogenic | POIKTMP | (1, 2, 53) |
| 18 | c.1884T>A (p. Ser628Arg) | Non-synonymous  Pathogenic | POIKTMP with Pancreatic cancer | (11) |
| 19 | c.1886T>G (p. Phe629Cys) | Non-synonymous  Pathogenic | POIKTMP with Pancreatic cancer with Liver Cirrhosis and Steroid Responsive Pneumonia | (4) |

? Not verified/may be associated with other disease-causing gene mutations.
